# Gender differences in outcomes after left atrial appendage closure with Watchman FLX device: insights from the Italian-FLX registry

**DOI:** 10.3389/fcvm.2024.1419018

**Published:** 2024-07-25

**Authors:** Michela Bonanni, Marco Frazzetto, Annalisa Nardone, Francesco Meucci, Carmine Musto, Gaetano Quaranta, Salvatore Saccà, Francesco Bedogni, Diego Maffeo, Fabrizio Ugo, Fabrizio Guarracini, Giacomo Bocuzzi, Alessandro Durante, Antonino Granatelli, Gabriele Tumminello, Geppina Eusebio, Carmelo Grasso, Federico De Marco, Bernardo Cortese, Massimiliano Mariani, Sergio Berti

**Affiliations:** ^1^ UOC Diagnostica Interventistica, Ospedale del Cuore Fondazione C.N.R. Regione Toscana G. Monasterio Massa, Massa, Italy; ^2^ Department of Biomedicine and Prevention, Policlinico Tor Vergata, Roma, Italy; ^3^ Division of Cardiology, A.O.U. Policlinico “G. Rodolico San Marco”, Catania, Italy; ^4^ Structural Interventional Cardiology, Department of Clinical and Experimental Medicine, Clinica Medica, Careggi University Hospital, Florence, Italy; ^5^ Division of Interventional Cardiology, Azienda Ospedaliera S. Camillo Forlanini, Rome, Italy; ^6^ Department of Cardiology, Umberto I° Hospital, Salerno, Italy; ^7^ Division of Cardiology, ULSS 3 Serenissima - Mirano Hospital, Mirano, Italy; ^8^ Department of Cardiology, IRCCS Policlinico San Donato, Milan, Italy; ^9^ Valve Center, Fondazione Poliambulanza Institute, Brescia, Italy; ^10^ SC Cardiologia, Ospedale Sant'Andrea di Vercelli, Vercelli, Italy; ^11^ Cardio-Thoraco-Vascular Department, Electrophysiology Unit, ASST Grande Ospedale Metropolitano Niguarda, Milan, Italy; ^12^ Division of Cardiology, San Giovanni Bosco Hospital, Turin, Italy; ^13^ Clinical and Interventional Cardiology Unit, Policlinico San Marco, Zingonia, Italy; ^14^ Interventional Cardiology, Sandro Pertini Hospital-ASL RM2, Rome, Italy; ^15^ Department of Cardio-Thoracic-Vascular Diseases, Foundation IRCCS Ca’ Granda Ospedale Maggiore Policlinico, Milan, Italy; ^16^ Division of Cardiology, Maria SS Addolorata Hospital of Eboli, Salerno, Italy; ^17^ Department of Cardiology, Monzino Cardiology Center, Milan, Italy; ^18^ Cardiovascular Research Center, Fondazione Ricerca e Innovazione Cardiovascolare, Milan, Italy

**Keywords:** gender differences, left atrial appendage occlusion, Watchman FLX, short and long term outcome, atrial fibrillation

## Abstract

**Introduction:**

Recent studies have shown gender differences in cardiovascular outcomes after left atrial appendage closure (LAAC), highlighting different complication rates and adverse events, particularly in short-term assessments. As a result, there remains a significant knowledge gap on how these differences directly impact the efficacy and safety of LAAC procedures. The aim of this retrospective study was to investigate the clinical outcomes of LAAC in women and men using the Watchman FLX device.

**Methods:**

This retrospective, multicenter study analyzes gender-specific outcomes in 650 patients who underwent LAAC with the Watchman FLX device between March 2019 and May 2022, drawn from the ITALIAN-FLX registry.

**Results:**

The results show comparable rates of all-cause mortality, stroke, transient ischemic attack and major bleeding in men and women 12 months after the procedure. Notably, no significant gender differences were found for periprocedural complications.

**Conclusion:**

In conclusion, this study shows that LAAC with the Watchman FLX device has comparable clinical outcomes between genders at both short-term and long-term follow-up.

## 1 Introduction

Left atrial appendage closure (LAAC) has been shown to be a safe and effective alternative to anticoagulant therapy for stroke prevention in patients with non-valvular atrial fibrillation (NVAF) and high bleeding risk or contraindication to anticoagulation (1–3). Several transcatheter techniques and devices for transcatheter LAAC have been developed over the last two decades. Gender disparities in cardiovascular disease have been widely documented and indicate differences in disease manifestations, treatment response and outcomes between men and women (4, 5). A comprehensive understanding of these differences is crucial for tailoring therapeutic approaches and achieving optimal outcomes even in male and female patients undergoing LAAC. Recent studies have highlighted the impact of gender difference of in-hospital safety outcomes following LAAC and have shown that women experience higher rates of adverse events such as pericardial effusion requiring drainage and major bleeding than men with the previously approved Watchman device (6, 7). The Amulet IDE trial is a randomized, controlled, non-inferiority trial comparing the Amulet Left Atrial Appendage Occluder with the Watchman device for stroke prevention (8). Long-term gender-specific outcome data after LAAC was analyzed in a post-hoc analysis of this study after 18 months of follow-up and showed no differences in ischemic stroke or systemic embolism, transient ischemic attack (TIA), major bleeding, hemorrhagic stroke, cardiovascular and all-cause death between men and women with both devices (9). Watchman FLX is the new generation device designed to overcome certain limitations of previous technology and to simplify implantation in a wider range of left atrial appendage (LAA) anatomies. Many observational studies showed a low incidence of adverse events and a high incidence of success closure with this device (10–12). Nevertheless, there is limited data on gender-specific outcomes after LAAC with the Watchman FLX device. The aim of this study is to investigate and analyze gender differences in short and long-term outcomes after LAAC with the Watchman FLX device in a real-life practice using data from the national multicenter ITALIAN-FLX registry.

## 2 Methods

**Figure 1:**
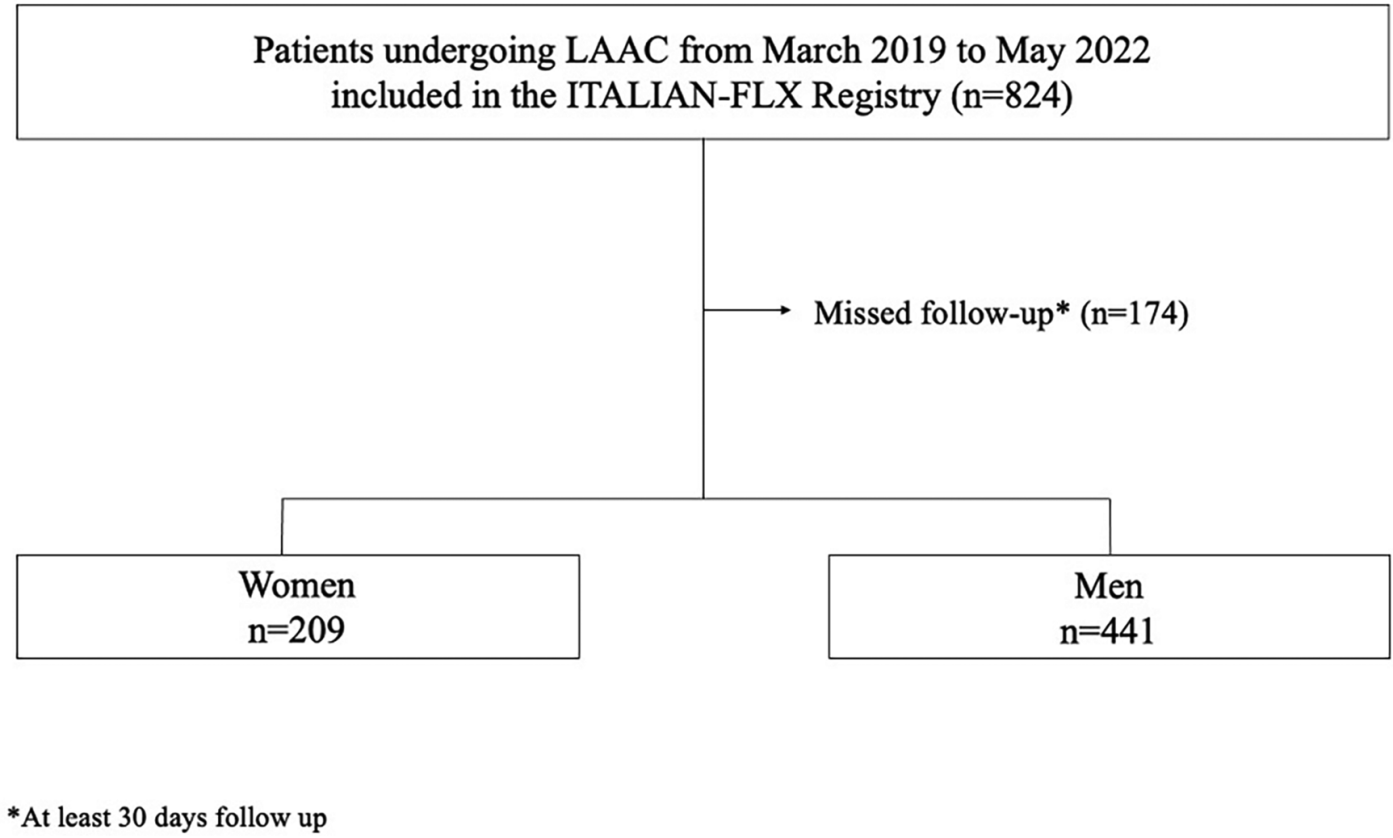
Study flow-chart.

The ITALIAN-FLX registry is a retrospective, non-randomized, multicenter study aimed to assess the periprocedural and long-term efficacy of the Watchman FLX device. The 25 study centers in Italy consecutively enrolled 824 patients undergoing LAAC with Watchman FXL device between March 2019 and May 2022. We excluded patients with-out follow-up data (Figure 1). Patients who had at least 30 follow-up days were included in the study. The peri-procedural results of the ITALIAN-FLX registry have been previously published (12). In this study, we sought to evaluate gender differences in terms of peri-procedural and long-term outcomes. Baseline clinical data such as gender, age, diabetes mellitus, hypertension, previous cardiovascular interventions and previous myocardial infarction (MI), stroke/transient ischemic attack (TIA), vascular disease, history of major bleeding, CHA2DS2-VASc score, HAS-BLED score, left ventricular ejection fraction (LVEF) were recorded for each patient. Orifice and length of the LAA were measured by transesophageal echocardiography (TOE). Periprocedural clinical outcomes, occurring 7 days after procedure, were reported as total events of all-cause death, stroke/TIA, major bleeding, MI, device embolization, pericardial effusion, pericardial tamponade and major vascular complications. Patients who had at least 30 follow-up days were included in the study. Long-term clinical outcomes were reported as total events of stroke or systemic embolism, TIA, major bleeding, minor bleeding, cardiovascular death (CV) death, all-cause death. Periprocedural outcomes and long-term clinical outcomes (up to 1 year) were compared between men and women. Events that occurred during the implantation procedure and within 7 days after the procedure were reported as periprocedural complications (6).

### 2.1 Study endpoints

The primary endpoint was the composite of cardiovascular death, stroke or systemic embolism, TIA and major bleeding events at 1 year. The co-primary endpoints were peri-procedural complications. Secondary endpoints were each single component of the primary endpoint, minor bleeding events and all-cause death. All endpoints were defined according to the the Munich Consensus, and the Bleeding Academic Research Consortium classification (BARC) (13–15).

### 2.2 Statistical analysis

Baseline clinical characteristics were summarized using descriptive statistics. Continuous variables were presented with means and standard deviations (SD) or median and interquartile range (IQR), while categorical variables were presented with counts (*n*) and percentages. Comparisons between groups were conducted using independent sample t-tests for normally distributed data and Mann–Whitney U tests for non-normally distributed data. Categorical data were compared using chi-square tests. Periprocedural clinical outcomes were determined using chi-square tests, with odds ratios (ORs) and 95% confidence intervals (CIs) calculated accordingly. Long-term clinical outcomes were analyzed with Kaplan-Meier method and compared using the log-rank test. Clinically relevant variables were evaluated as univariable predictors of 1-year primary composite endpoint using Cox proportional hazards regression models. A subset of the clinically relevant univariable predictors with *P* ≤ 0.10 were selected for inclusion in a multivariable model using the stepwise method with threshold *P* = 0.10 for entry and exit. Regression models are summarized as hazard ratios and 95% CIs. Results are considered significant if *P* < 0.05. All data were analyzed with SPSS version 29 (IBM software).

## 3 Results

### 3.1 Baseline patient characteristics

Of 650 patients included in our study, 209 (32.2%) were women and 441 (67.8%) were men. Compared to men, women were older (77.8 ± 7.3 vs. 76.1 ± 8.4, *p* = 0.012), less likely to have had previous myocardial infarction (18.2% vs. 26.5%, *p* < 0.02), previous cardiovascular procedure (26.3% vs. 47.8%, *p* < 0.003), carotid disease (7.7% vs. 15.6%, *p* < 0.005), and with higher LVEF [56 (50–60) vs. 53 (45–60), *p* < 0.05]. Compared to men, women had higher proportion of paroxysmal atrial fibrillation (41.6% vs. 33.8%, *p* = 0.048). Among woman, 71.8% had hypertension, 28.7% had diabetes mellitus, 39.6% had hematologic disorders, 18.2% had a history of ischemic stroke/TIA history and 28.7% had previous gastrointestinal bleeding. These clinical characteristics did not differ significantly between the two groups. Compared to men, women had fewer previous central nervous system bleeds (8.6% vs. 13.6%, *p* = 0.067), a lower HAS-BLED score (3.5 ± 1.1 vs. 3.8 ± 1.1, *p* < 0.001) and a higher average of CHA2DS2-VASc score (4.6 ± 1.3 vs. 3.9 ± 1.4, *p* < 0.001). There were no significant differences between the groups in either BMI or permanent/persistent AF. Measurements of LAA orifice and length performed by TEE were similar in women and men. Table 1 shows patients baseline characteristics.

**Table 1:** Baseline patient characteristics.

Characteristics	Overall (*n* = 650)	Men (*n* = 441)	Women (*n* = 209)	*p*-value
Age, yrs	76.7 ± 8.1	76.1 ± 8.4	77.8 ± 7.3	0.012
Diabetes	200 (30.7)	140 (31.7)	60 (28.7)	0.433
Hypertension	498 (76.5)	348 (78.9)	150 (71.8)	0.108
BMI, kg/m^2^	26.4 ± 4.3	27 ± 3.5	26.4 ± 4.3	0.084
Atrial fibrillation				
Permanent	322 (49.5)	228 (51.7)	94 (45)	0.115
Persistent	79 (12.1)	55 (12.5)	24 (11.5)	0.743
Paroxysmal	235 (36.1)	149 (33.8)	87 (41.6)	0.048
Prior stroke/TIA	220 (33.8)	157 (35.6)	63 (30.2)	0.172
Myocardial infarction	155 (23.8)	117 (26.5)	38 (18.2)	0.020
LVEF, %	55 (45–60)	53 (45–60)	56 (50–60)	<0.000
Cardiovascular procedure	266 (40.8)	211 (47.8)	55 (26.3)	0.003
Carotid diseases	85 (13.1)	69 (15.6)	16 (7.7)	0.005
eGFR, ml/m^2^	59 (43.9–69.5)	59 (45.1–72.5)	59 (42.9–62.7)	0.008
Cancer	61 (9.4)	39 (8.8)	22 (10.5)	0.492
Haematologic disorders	65 (10)	45 (10.2)	20 (9.6)	0.801
CHA2-DS2-VASc score	4.6 ± 1.3	3.9 ± 1.4	4.6 ± 1.3	<0.001
Major bleeding				
Gastrointestinal	196 (30.1)	136 (30.8)	60 (28.7)	0.606
CNS	78 (12)	60 (13.6)	18 (8.6)	0.067
HAS-BLED score	3.5 ± 1.1	3.8 ± 1.1	3.5 ± 1.1	<0.001
Very high bleeding risk	89 (13)	52 (11.8)	37 (17.7)	0.041
LAA orifice by TEE, mm	19.2 ± 6.6	19.5 ± 6.3	18.6 ± 7.3	0.352
LAA length by TEE, mm	25.6 ± 9.4	26.1 ± 9	24.4 ± 10	0.153

BMI, body mass index; TIA, transient ischemic attack; LVEF, left ventricular ejection fraction; eGFR, estimated glomerular filtration rate; LAA, left atrial appendage; TEE, transesophageal echocardiography, IQR, interquartile range. Values are mean ± SD, median (interquartile range), or *n* (%).

### 3.2 Procedural characteristics

The procedural characteristics showed no significant differences between the two groups. There were no gender significant differences in median procedure duration, amount of contrast agent used, or type of procedural guidance used. Table 2 shows procedural characteristics.

**Table 2:** Procedural characteristics.

Characteristics	Overall (*n* = 650)	Men (*n* = 441)	Women (*n* = 209)	*p*-value
Procedure time, min	57 (38–75)	55.5 (37–75)	59 (40–79.5)	0.665
Contrast use, ml	92.31 (60–110)	89.83 (60.00–102.50)	97.81 (50.00–116.25)	0.909
Procedural guidance				
TEE	526 (80.9)	357 (81)	169 (80.9)	0.528
ICE	133 (20.5)	89 (20.2)	44 (21.1)	0,436
Size of device				
FLX 20	82 (12.6)	61 (13.8)	21 (10.0)	0.206
FLX 24	165 (25.4)	105 (23.8)	60 (28.7)	0.210
FLX 27	192 (29.5)	137 (31.1)	55 (26.3)	0.232
FLX 31	133 (20.5)	89 (20.2)	44 (21.1)	0.835
FLX 35	78 (12.0)	52 (11.8)	26 (12.4)	0.797

Values are mean ± SD, median (interquartile range), or *n* (%). ICE, intracardiac echocardiography; TEE, transesophageal echocardiography.

### 3.3 Peri-procedural complications

No difference was found between the groups with regard to procedural complications (Table 3). The rate of in-hospital adverse events, which included ischemic stroke, TIA, pericardial tamponade, major and minor bleeding, major vascular complications, and pericardial effusion, showed no difference between men and women. In addition, no device embolization or myocardial infarctions were reported in either group. In-hospital death occurred rarely and were comparable between women and men (0.5% vs. 0.2%; *P* = 0.540).

**Table 3:** Periprocedural complications.

Complications	Overall (*n* = 650)	Men (*n* = 441)	Women (*n* = 209)	*p*-value
Death	2 (0.3)	1 (0.2)	1 (0.5)	0.540
Ischemic stroke	2 (0.3)	2 (0.5)	0 (0.0)	0.460
Transient ischemic attack	1 (0.2)	0 (0.0)	1 (0.5)	0.322
Major bleeding	14 (2.1)	9 (2.0)	5 (2.3)	0.536
Minor bleeding	8 (1.2)	6 (1.4)	2 (1.0)	0.497
Myocardial infarction	0 (0.0)	0 (0.0)	0 (0.0)	
Device embolization	0 (0.0)	0 (0.0)	0 (0.0)	
Pericardial tamponade	2 (0.3)	1 (0.2)	1 (0.5)	0.540
Major vascular complication	19 (2.9)	12 (2.7)	7 (3.3)	0.627
Length of hospital stay	3.8 ± 2.3	3.6 ± 2.3	4.1 ± 2.4	0.038

Values are *n* (%) of patient group using chi-square test.

### 3.4 Secondary endpoints

**Figure 2:**
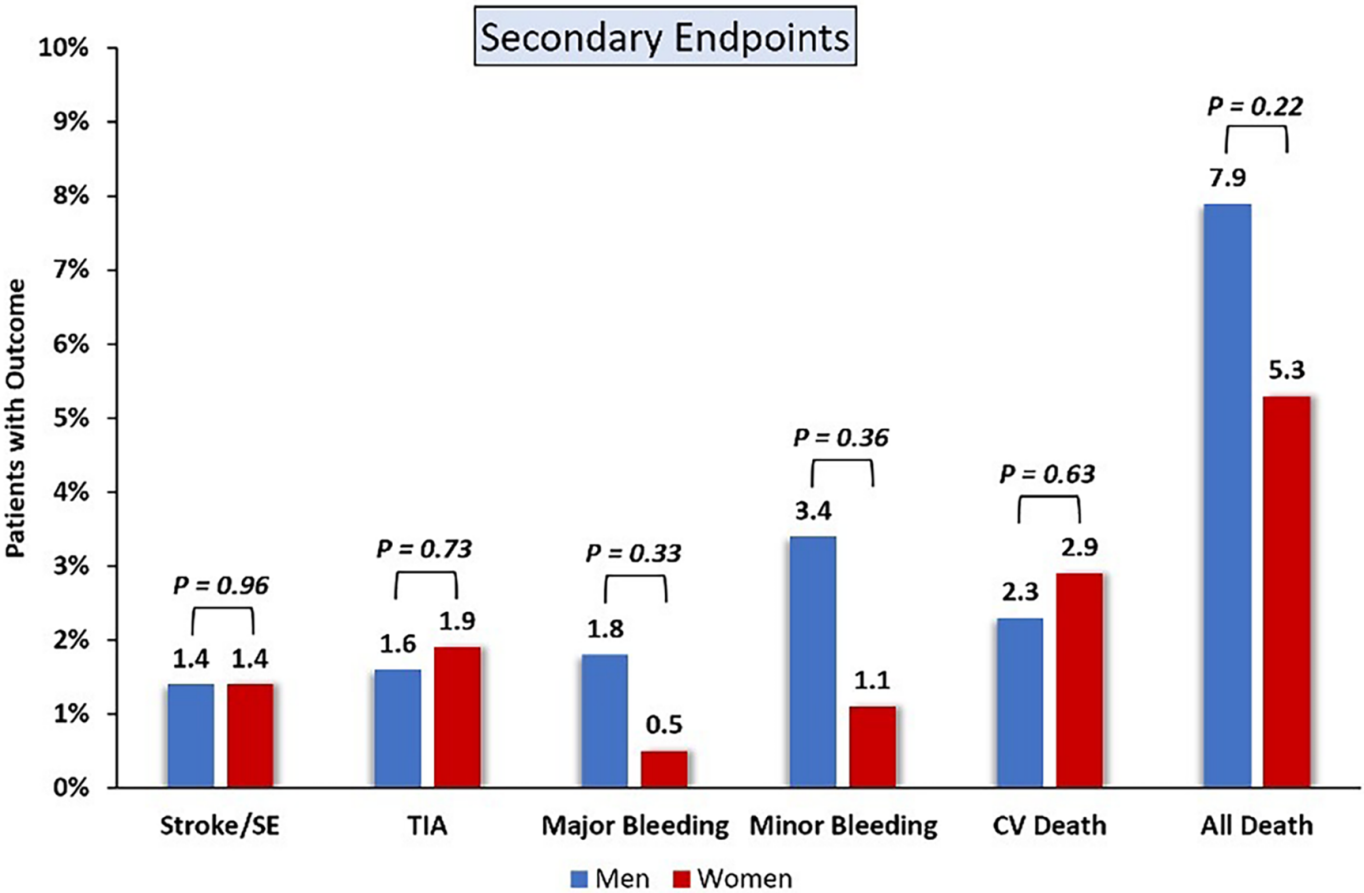
Secondary endpoint.

The average follow-up time was 354 days. A total of 16 patients, including 6 women and 10 men, died of cardiovascular death. Nine cases of ischemic stroke were recorded, including 3 women and 6 men, while 11 patients (7 men and 4 women) suffered from TIA. Fifteen patients, including 3 women and 12 men, experience major bleeding. A total of 29 patients (22 men and 7 women) experienced minor bleeding. There were no significant differences between women and men in terms of death from cardiovascular disease (2.9% vs. 2.3%; *P* = 0.630), ischemic stroke and TIA (1.4% vs. 1.4%; *P* = 0.960; 1.9 vs. 1.6%, *P* = 0.726), major bleeding (0.5% vs. 1.8%; *P* = 0.327) and minor bleeding (1.1% vs. 3.4%; *P* = 0.360), or all-cause death (5.3% vs. 7.9%; *P* = 0.225) (Figure 2). Table 4 shows secondary endpoints.

**Table 4:** Secondary endpoints.

secondary endpoints	Overall (*n* = 650)	Men (*n* = 441)	Women (*n* = 209)	*p* log-rank
Stroke or systemic embolism	9 (1.38)	6 (1.4)	3 (1.4)	0.960
TIA	11 (1.7)	7 (1.6)	4 (1.9)	0.726
Major bleeding	15 (2.3)	12 (1.8)	3 (0.5)	0.327
Minor bleeding	29 (4.5)	22 (3.4)	7 (1.1)	0.360
CV death	16 (2.5)	10 (2.3)	6 (2.9)	0.630
All cause death	46 (7.2)	35 (7.9)	11 (5.3)	0.225

Values are *n* (%) of the patient group using the Kaplan-Meier method. Events occurring prior to hospital discharge date were excluded from this analysis. TIA, transient ischemic attack; CV, cardiovascular.

### 3.5 Primary composite endpoint

**Figure 3:**
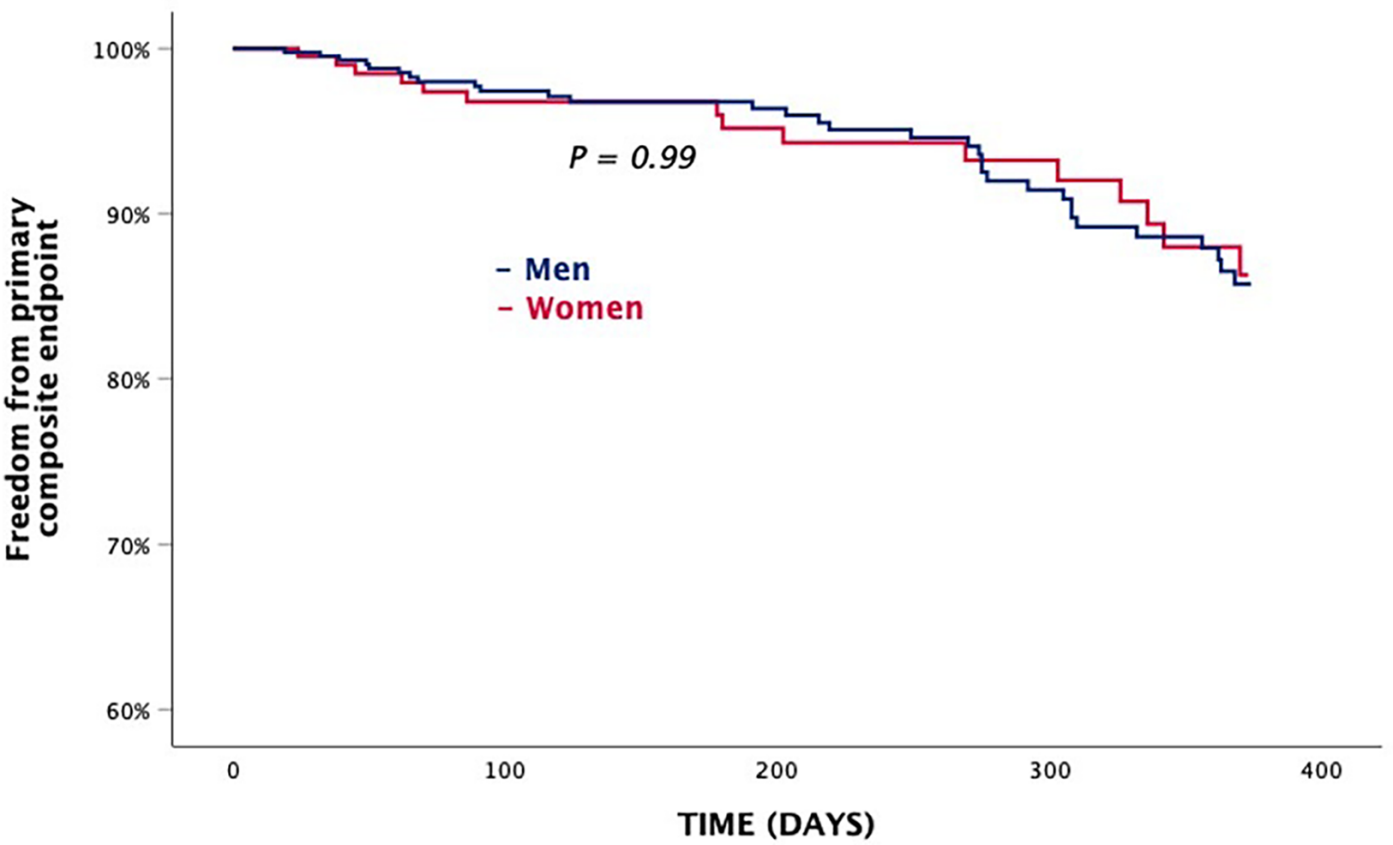
Primary composite endpoint.

Kaplan–Meier analyses showed that survival of the primary composite endpoint was comparable between groups (Figure 3). Clinically significant baseline predictors of the primary composite endpoint were diabetes [HR, 2.04 (CI, 1.13–3.68); *P* < 0.018], left ventricular ejection fraction [HR, 0.97 (CI, 0.95–0.99); *P* < 0.015], and prior stroke [HR, 2.21 (CI, 1.09–4.48); *P* < 0.027] (Table 5).

**Table 5:** Univariable and multivariable predictors of primary composite endpoint.

Predictors	Univariable		Multivariable	
	Hazard ratio (95% CI)	*P*-value	Hazard ratio (95% CI)	*P*-value
Coronary artery disease	2.06 (1.15–3.70)	0.015	1.33 (0.70–2.52)	0.383
eGFR, ml/min	0.98 (0.97–1.00)	0.06	0.99 (0.98–1.01)	0.347
Previous stroke	2.27 (1.13–4.58)	0.022	2.21 (1.09–4.48)	0.027
Diabetes	2.33 (1.31–4.13)	0.004	2.04 (1.13–3.68)	0.018
LVEF, %	0.96 (0.94–0.98)	<0.001	0.97 (0.95–0.99)	0.019
Hypertension	1.24 (0.62–2.46)	0.539		
BMI, kg/m^2^	0.98 (0.90–1.06)	0.581		
Age, year	1.00 (0.97–1.04)	0.863		
HAS-BLED score	1.25 (0.96–1.63)	0.971		

Variables in the multivariable model were selected from univariable predictors with *P* value ≤0.10. LVEF, left ventricular ejection fraction; eGFR, estimated glomerular flow rate; BMI, body mass index.

## 4 Discussion

The main findings of the study are as follows: (1) at 1 year post LAAC, there was comparable rate of primary composite endpoint observed between men and women; (2) no significant gender differences were noted in peri-procedural complications; (3) there were no disparities in the secondary endpoints: stroke or systemic embolism, TIA, major bleeding, minor bleeding and CV death.

The study contrasts with previous research indicating a higher incidence of complications in women compared to men, particularly major bleeding, pericardial effusions requiring intervention and vascular complications (6, 9, 16–18). However, the results of this study are consistent with those of De Caterina et al. and Zhao et al. who reported no gender difference in perioperative outcomes (19, 20).

In our study, the comparable incidence of in-hospital complications between groups suggests that LAAC may offer similar effectiveness regardless of gender. This result could be attributed to the innovative features of the Watchman-FLX device, such as enhanced conformability, a flattened surface, reduced length, and improved maneuverability. Additionally, most operators were experienced in using devices with a single occlusive mechanism. Furthermore, the utilization of a singular device helped to minimize any potential gender-device interactions, thereby potentially enhancing the uniformity of outcomes observed across both genders in the study.

In terms of long-term outcomes, the results of a sub-analysis of the Amulet IDE trial, which analyzed gender differences in patients undergoing LAAC, found no differences in ischemic events or mortality between genders at 18 months (9). However, it is noteworthy that women had a higher incidence of hemorrhagic events, especially major bleeding during hospitalization (9). The sub-analysis of the Amulet observational study, which was conducted on 1,099 patients, confirmed these results and showed no difference in primary outcomes between men and women after 2 years of follow-up (19). Notably, Chen et al. reported similar results in cardiovascular or unexplained death, major bleeding and ischemic stroke, fatal or disabling ischemic stroke at 4.3 years follow-up (21). A meta analysis, including three studies examined the long-term mortality rates of patients undergoing LAAC, did not find a significant association between sex and long-term mortality risk (18). In line with these data, our study showed no differences in cardiovascular death, stroke or systemic embolism, TIA and major bleeding at 1 year follow-up, reinforcing that LAAC may indeed be equally effective in both genders in terms of preventing adverse cardiovascular events over the long term. This is the first study analyzing gender differences in short and long term outcome after LAAC procedure with Watchman FLX device. Important strengths of our study are the large sample size and the use of a single device, so that potential gender-specific interactions between device type and periprocedural complications could be excluded, enhancing the reliability of the findings. This study has some limitations. First, study results could be affected by disparities in missing data and completion of follow-up visits. Second, the majority of patients were only followed up for 10 months, although a longer follow-up is ongoing. Third, the study did not report data on medical therapy, which could influence outcomes. Fourth, smaller sample size compared to other studies, may not be sufficient to detect small gender differences given the low adverse event rate of this procedure.

In conclusion, this study provides valuable insight into gender differences in short- and long-term outcomes following LAAC procedures using the Watchman FLX device. While the results suggest similar efficacy in both genders, further research and longer-term follow-up are needed to fully understand the impact of LAAC on cardiovascular outcomes in different patient groups. The LAAC procedure, performed by experts, has a low complication rate, even when performed in a real-world population at high risk of events.

## Data Availability

The raw data supporting the conclusions of this article will be made available by the authors, without undue reservation.
